# Federated blockchain system (FBS) for the healthcare industry

**DOI:** 10.1038/s41598-023-29813-4

**Published:** 2023-02-13

**Authors:** Ashraf Mohey Eldin, Eman Hossny, Khaled Wassif, Fatma A. Omara

**Affiliations:** Department of Computer Science, Faculty of Computers and Artificial Intelligence, Cairo University, Cairo, Egypt

**Keywords:** Health services, Computer science

## Abstract

Blockchain is a distributed technology that introduced the well known Bitcoin cryptocurrency into action. Blockchain has been considered for research by many countries and industries. It is being applied in many fields such as the healthcare domain. Many companies started using Blockchain to increase the security and privacy of the Electronic Healthcare Records for their patients. The work in this paper discusses some existing healthcare problems and challenges. In addition, the paper reviews some related work models and provides a comparison that shows their objectives and limitations. Also, a proposed Federated Blockchain System (FBS) is introduced to provide solutions for these healthcare problems and elaborates the technical details of the system architecture. Moreover, the effectiveness of the system has been validated which showed an average of 68–100 ms for performing query operations and average of 0.944–19.041 s for performing writing operations on the system. Finally, a discussion of the system validation and future work are presented.

## Introduction

Bitcoin was introduced to allow two parties to transfer digital cryptocurrency assets between each other without any central authority^1^. In addition, Bitcoin introduced the blockchain technology which caused a revolution in the distributed systems. The blockchain is basically a distributed database including blocks of all transactions or events that have been executed. These blocks are being shared among all entities that are participating in the blockchain network^2^. Every transaction is verified by the majority of nodes before being added to the chain. Each block contains the hash of the previous block. Thus, tampering a block on a chain will change its hash and break the whole chain which makes the blockchain immutable.

Blockchain has been adopted by many domains, such as banking, supply chain management, and healthcare^3^. The work in his paper focuses on blockchain for the healthcare domain. The healthcare industry has been suffering from many security issues related to EHRs management. One of these problems is providing a unique identifier for the patient to be able to track the patient’s medical history across multiple healthcare organizations^4^. Also, having a mechanism that enables sharing of the medical history of the patient among different healthcare organizations has been a fatal problem^5^. Recently, researchers have started to consider blockchain technology to solve these healthcare issues. Researchers invented many models such as MedChain^6^, InterChain^7^ and ShareChain^8^ that aim to provide a mechanism for data sharing of patient’s medical records. Unfortunately, some of these invented models remain as novel models without any implementation yet. A detailed discussion of these models and their limitations can be found in “Related work”.

The work in this paper discusses the healthcare problems and introduces a new system that aims to provide a scalable federated healthcare blockchain system which solves the patient's unique identity challenge and provides an interoperability mechanism among different healthcare organizations. In addition, the base implementation of that system is provided along with system validation analysis. Finally, the future work is discussed.

The rest of the paper is organized as follows; “Background” introduces a background about the healthcare industry, how it can grow, and the issues that are facing healthcare organizations regarding EHRs security and management. In “Related work”, the discussion of related work is provided along with their limitations. Then, “Proposed federated blockchain system (FBS)” introduces the technical details of the proposed system to overcome these healthcare EHRs issues. The system validation analysis and limitations of the system are discussed in “System validation”. Finally, “Conclusion and future work” concludes the paper and provides future work discussion.

## Background

Healthcare providers are seeking to improve the quality of the healthcare service. The improvement techniques are divided into vertical and horizontal directions^9^. The vertical direction is improving fast as new medicines are being invented every day. However, the horizontal improvements are happening at a slow rate because many healthcare providers take a lot of time to change the way they operate. The blockchain is considered a horizontal technology for healthcare. We believe applying blockchain in the healthcare domain can improve the quality of the healthcare services.

The healthcare organizations suffer from many problems including the following challenges:

### Patient’s medical history

One of the biggest problems that are facing the healthcare industry is that there is no consistent way to keep track of the patient’s medical history. A lot of EHRs get lost when the patient moves from one hospital to another. Actually the problem is not only about lost EHRs, but also about miscommunication of EHRs from one healthcare organization to another which can endanger the patient’s life as he may receive a medical treatment which is not suitable for his medical case^10^.

### Patient’s unique identifier (ID)

Another issue that is facing the healthcare industry is that there is no unique ID for each patient. When the patient goes to a hospital to receive treatment, the hospital creates an entry for him on its system to be able to record his medical reports under this entry. When the patient goes to another hospital, another entry is created for him on the hospital’s system to record his medical reports. Eventually, the patient will have multiple IDs for each hospital. The issue can go worse where a single hospital may create multiple entries for the same patient, so his medical records will be distributed across two or more IDs inside the same hospital system. That will cause a lot of conflicts which in turn will contribute to the lost EHRs issue as mentioned above^4^.

### Interoperability and data blocking

One of the biggest problems that are facing the healthcare industry is the interoperability problem. After the patient receives his medical treatment at a specific hospital, that hospital tends to keep his data away from other hospitals to guarantee that the patient will continue to receive medical treatment there. This issue is known as data blocking in which a healthcare organization does not want to share or communicate patient data to another healthcare organization. In addition, there is no unified mechanism that facilitates EHRs from one organization to another. This problem also contributes to the patient’s medical history issue^5^.

### Medical records security

Medical records are very sensitive data that should be stored in a secure way. It should be kept away from attackers. Unfortunately, many traditional hospital systems suffered from data breaches over the last two decades. According to The Department of Health and Human Services, during 2021 more than 500 data breaches occurred during which more than 44,993,618 healthcare records have been exposed or stolen^4^.

### Medical record authenticity and integrity

Medical records must be verified before being recorded on the system. The ownership of the EHRs must be guaranteed to a specific patient. Thus, EHRs must be authentic. Also, EHRs must not be altered by any unauthorized party. Attacks that can modify or add malicious data to the patient’s EHRs will definitely put the patient’s life in danger^11^.

## Related work

In this section, we will review some blockchain models that can be applied to the healthcare industry. Some of them are proposed models without implementation yet. They address the problems that challenge healthcare organizations and how they can be solved using blockchain.

### A decentralized privacy-preserving healthcare blockchain for IoT

**Figure 1:**
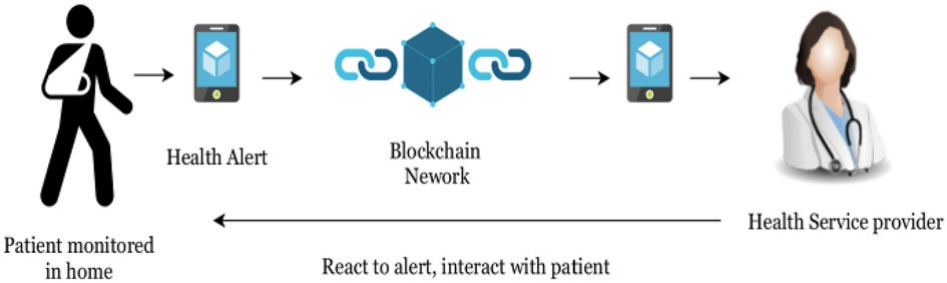
Remote patient monitoring^12^.

In this model, the authors are proposing a modified blockchain that handles the transactions of the medical IoT devices in a secure way^12^. These devices are connected to patients to monitor some measures such as sugar level and then send the collected EHRs over the modified blockchain to a healthcare organization which reacts to them as shown in Fig. 1. The collected EHRs could endanger the patient’s life if a delay happens or if these data are changed in some way.

The model enhances the security and privacy of the transaction using advanced cryptographic primitives. It also eliminates the huge computational power required by the blockchain and replaces it with a less resource intensive mechanism which is suitable for IoT devices.

The model aims to make the transactions on the network anonymous unlike Bitcoin in which everyone on the network has a public address and anyone can see what funds exist at this address^1^. Thus, they propose a lightweight privacy-preserving ring signature scheme to make transactions anonymous.

They also use a lightweight digital signature to make sure that the information is not tampered on the network and that it is signed by the actual sender. Also, they use light operations for the signature process to make it suitable for IoT.

Additionally, they use a double encryption mechanism to add an extra layer of protection for the medical data. This mechanism relies on encrypting the data first using a symmetric key, then encrypting the symmetric key using a public key. All of this is done using ARX cipher class which makes the encryption process suitable for IoT devices.

Since the blockchain contains many nodes that represent the IoT devices, It will suffer from scalability issues. However, the authors have removed the heavy computational PoW mechanism. They also divide the blockchain into clusters. Each cluster contains a set of nodes. Therefore, not all nodes will be in a single blockchain. Each cluster will be responsible for its nodes and the clusters will be linked together to transfer the data between each other using the cluster head which exists in each cluster.

The medical data may be large in size and storing it on the blockchain directly might not be possible. Therefore, the model suggests storing large medical data on secure cloud storage. However, the hash of the data will be always recorded on the blockchain network so that any tampering happens on the cloud storage will be detected by the blockchain network.

The model also uses smart contracts which are self-executing commands that fire when a specific event happens on the network. They use smart contracts to perform pre-coded actions. For example, an alarm will be sended when the blood pressure readings go above or below a predefined threshold value. The use of smart contracts in this way could help save patients’ lives.

The suggested modified blockchain model is private blockchain which uses PoA. Every patient provides identity documents and goes into a preliminary process before joining the network. Once approved, the patient can participate in the network.

As the case with any private blockchain model, this model suffers from some centralization problems. In addition, the blockchain is divided into clusters and each cluster has a cluster head node which may go offline for any reason and that will block its whole cluster until it goes online again. Also, the model does not have any implementation and cannot be evaluated yet.

### MedChain

**Figure 2:**
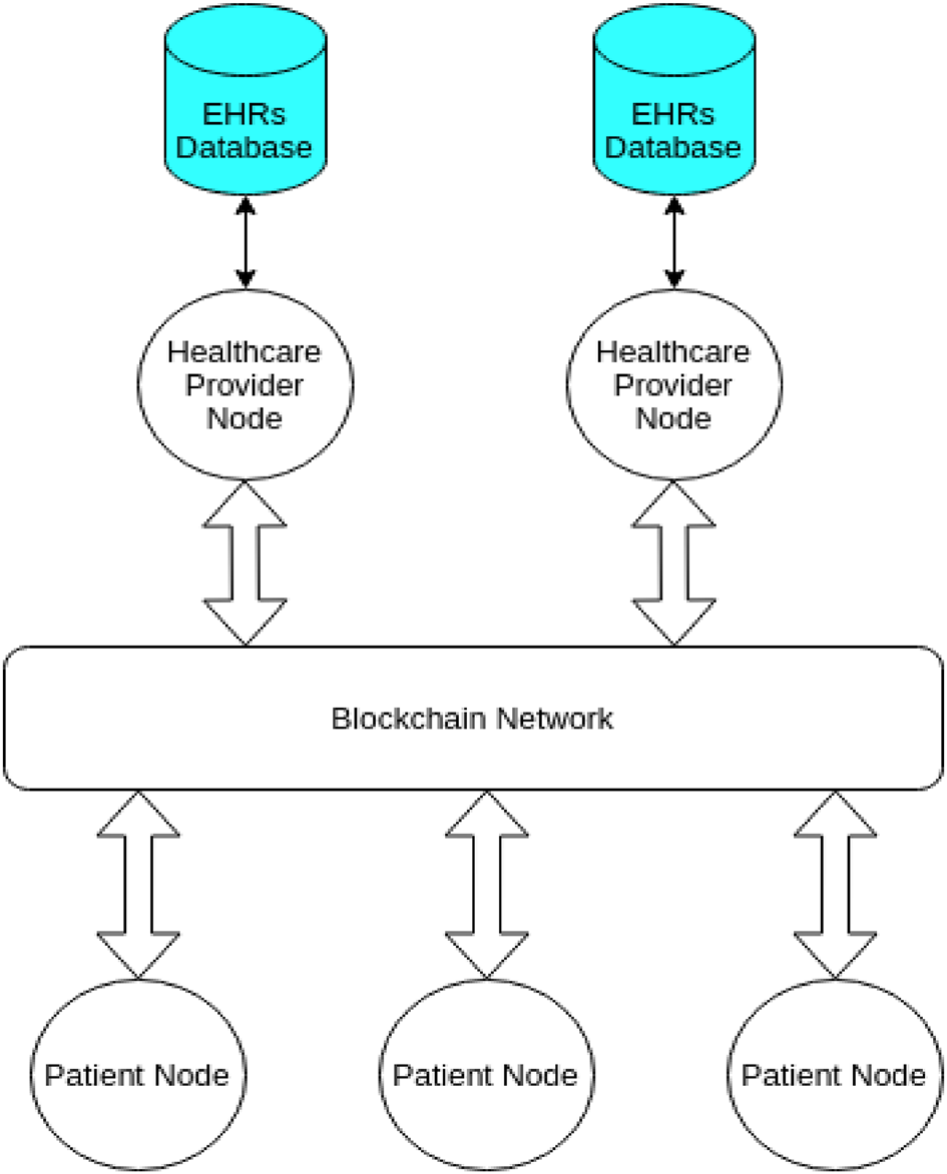
A simplified architecture of MedChain platform.

This model was invented to facilitate interaction between healthcare providers and patients for sharing data in a secure way while keeping the data private^6^. They modify the regular blockchain to make it suitable for large data sets with low transaction latency. The model records all access logs to the patients’ EHRs to have a full view of everything going inside the network. Healthcare provider nodes are responsible for maintaining their patients’ data as shown in Fig. 2. However, patients can query their records from the providers or give access to third parties for these records. The system treats the EHRs as assets which belong to the patients who own them.

The authors use the PoA consensus mechanism. They assign some degree (reputation) to the provider nodes and this degree increases based on the performance of the provider node for maintaining the patient’s records. Nodes with less degree are more likely to be selected to produce the next block, while nodes with higher degree will vote for the validity of the block. This will decrease the possibility of fraud nodes to join the network and will increase fairness among provider nodes for creating blocks.

The system also uses the distributed ElGamal re-encryption schema with distributed blinding to share EHRs record from one provider to another without sharing the credentials information for decrypting the record [34].

The healthcare providers can integrate to this platform using their existing database of records. They provide a mechanism and API written in GoLang that helps healthcare providers migrate to the system and create necessary links and hashes for the data queries then store them on the blockchain itself.

The authors also use a special set of smart contracts called timed smart contracts which help the system monitor the transactions and decide voter nodes and provider nodes that will create the block and ensure the integrity of the blocks. Additionally, these smart contracts govern access control over the network.

The platform is under development, but it is still in its early stages. However, according to the analysis done to this model, it is believed to provide the healthcare industry with very promising results. The platform aims to put control into patients’ hands which sometimes might not be the best solution.

### Integrating blockchain for data sharing and collaboration in mobile healthcare applications

The authors propose a mobile controlled model^13^. This model describes a secure method for healthcare data sharing between different medical institutions using blockchain. Also, the model aims to give patients more control for their EHRs. The model takes advantage of blockchain privacy-preserving property to secure patients identity while sharing their healthcare records^14^.

**Figure 3:**
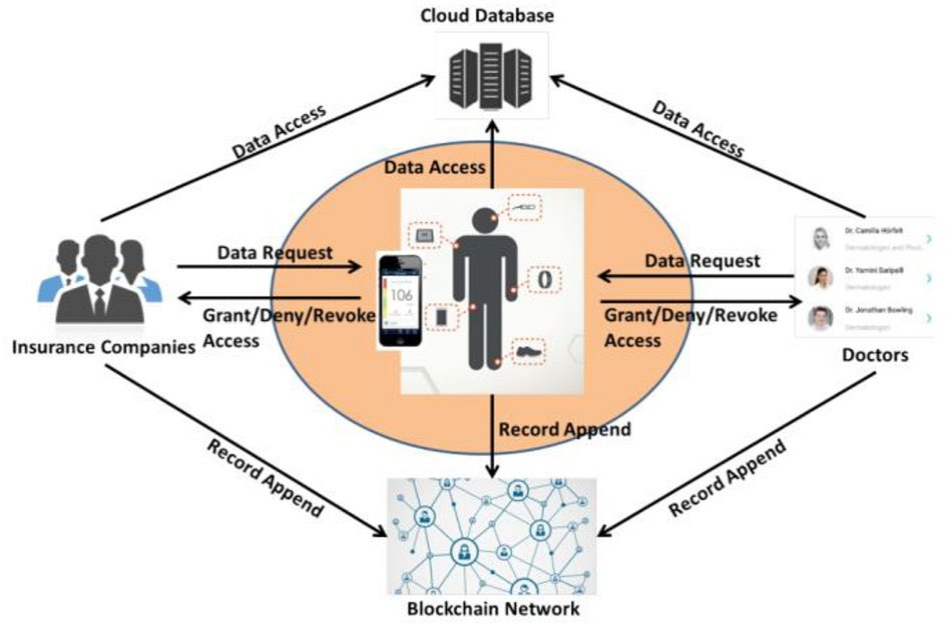
A user centric model^13^.

The authors propose using a mobile application from which the patient can grant, deny or revoke access to his data for some healthcare provider. They also use cloud storage to store EHRs that are collected from wearable devices or entered manually by doctors, healthcare providers or the patient himself as shown in Fig. 3.

The blockchain is used in this model to store the hash of the medical records that are being stored on the cloud. Additionally, the blockchain stores access control policies and stores access activity logs so they can track malicious activities on the network.

**Figure 4:**
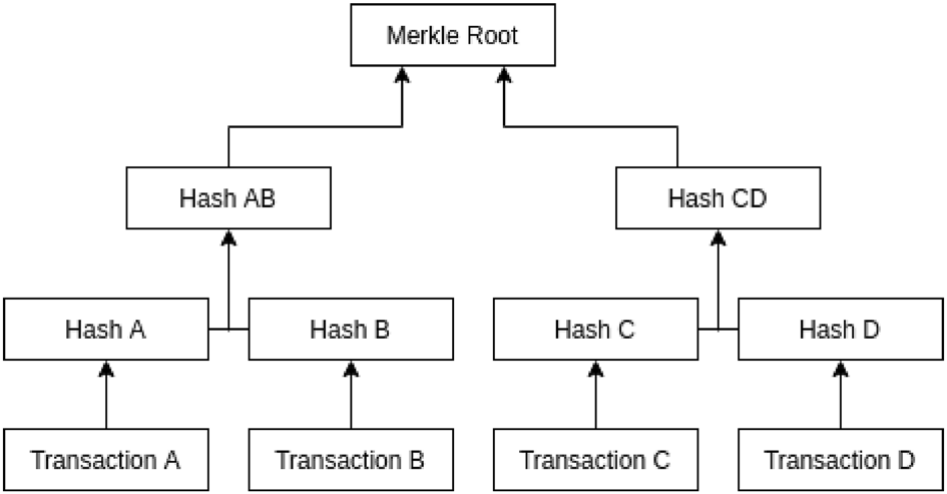
The generated Root hash from Merkle tree where Transaction A, B, C and D represents some EHRs.

They use a permissioned blockchain built on the Hyperledger Fabric platform to take advantage of its membership service component to manage access control for the data^15^. The blockchain also uses proof of integrity to validate data. Since there will be a huge number of EHRs to be stored, the authors propose using the Merkle tree-based technique for validating data integrity^16^. The Merkle tree improves the scalability of the system. Each Merkle tree can hold the hash of a list of EHRs and finally they store the Merkle root value (root hash) in the blockchain network as shown in Fig. 4. The integrity can be validated by traversing the tree and comparing the root hash with the one that exists in the blockchain. Any modification to the data will be easily detected as the hash value will not match the stored one.

The model gives the patient the option to add records to his data manually. This could be a point of entering fraud data especially for uneducated patients. Also, the model did not fully discuss the synchronization step between the cloud storage and the blockchain network. Besides that, this model has no actual implementation yet.

### IoT data privacy via blockchains and IPFS

**Figure 5:**
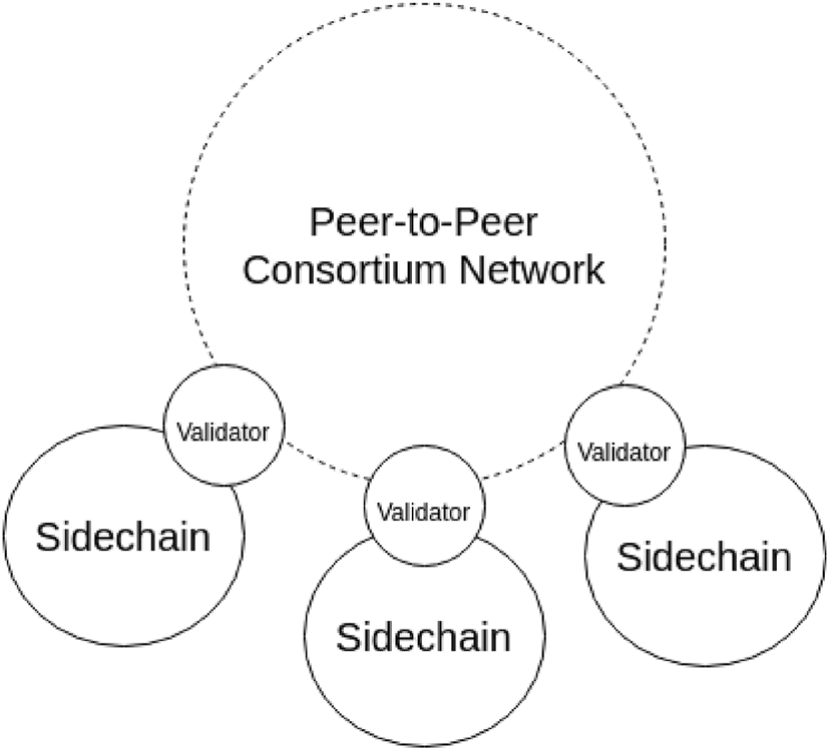
Sidechains connected to the peer-to-peer consortium network^17^.

This model is concerned about connecting IoT devices together via blockchain networks to interact with each other and store collected data^17^. It is a generic model that can be applied to the healthcare industry that uses IoT medical devices attached to patients to collect EHRs and send them. In this model, the authors propose modular consortium architecture to facilitate IoT devices communication and data sharing using blockchain networks. The authors discuss the privacy issues of IoT data using the existing client server model. The proposed model aims to give control to the user (the owner of collected data from IoT sensors) for his data without the need of a trusted centralized node to avoid any misuse of the user’s data. The model logs all transactions including data access. The IoT devices are grouped into sidechains which are private blockchains. Every user may have multiple sidechains containing his IoT devices. Every sidechain is responsible for recording all data logs. The sidechains are eventually linked together in a modular way using a decentralized peer-to-peer consortium network as shown in Fig. 5.

This consortium network is a higher level blockchain that handles incoming access requests for IoT collected data. The sidechains are made as private blockchain to overcome scalability and transaction delay problems. Users can have as many sidechains as required and the IoT devices inside these private sidechains are trusted, so there is no need to validate the transactions that happen which increases transaction rate. Also, the consortium network is responsible for logging access requests. The main reason for this separation is keeping the log of IoT devices data creation away from the log of data access so that all nodes inside the consortium network can see the access log, but nodes inside each sidechain can only see the data log making it more private and secure.

The sidechain contains a node called the validator node which is responsible for approving and writing blocks of data to the sidechains. These blocks of data are collected from different IoT devices inside the sidechain. Also, the sidechains can contain smart contracts depending on the implementation and the use case of the sidechain. The IoT devices within the sidechain can send data to the smart contracts using their addresses. The smart contracts are also used to manage access control for incoming requests. Additionally, the public address of all IoT devices of the sidechains are stored into smart contracts to prevent fraud transactions from unauthorized parties to be recorded inside the private sidechain.

The validator nodes of each sidechain are connected together to form a peer-to-peer consortium network.

This consortium network has also a smart contract that contains the address of the devices which are allowed to access certain data. So, the role of smart contracts in this layer is to regulate the access control coming from external requesters. To access data on this model, the external requester joins the consortium network and the owner of the sidechain adds the address of that requester to the smart contract’s address list on the sidechain.

Also, the authors have eliminated the use of PoW consensus mechanism and replaced it with PoS to overcome the computational power waste. The Proof of Stake is applied in the consortium blockchain network.

This model can be further adjusted to fit the healthcare industry. For instance, each hospital or healthcare organization can hold one or more sidechains. These sidechains are then connected together using the consortium networks which can be regulated by higher level healthcare organizations.

Similar to Ref.^12^, each sidechain has only one validator node since it is a private blockchain which makes it prone to point of failure and centralization issues. This could be improved by converting the private sidechain to a consortium sidechain that has multiple validator nodes which increases system availability and makes it a more trusted system. Also, this model is still novel and has no actual implementation yet.

### ShareChain

This framework provides a safe and secure atmosphere for sharing the patients’ EHRs to data consumers securely while keeping the identity of patients anonymous^8^. It is based on blockchain, local differential privacy (LDP), and federated learning (FL). In addition, it uses an Interplanetary file system (IPFS) to secure the data in the distributed system. The framework uses permissioned blockchain to avoid transaction latency issues and to increase the security of the network.

**Figure 6:**
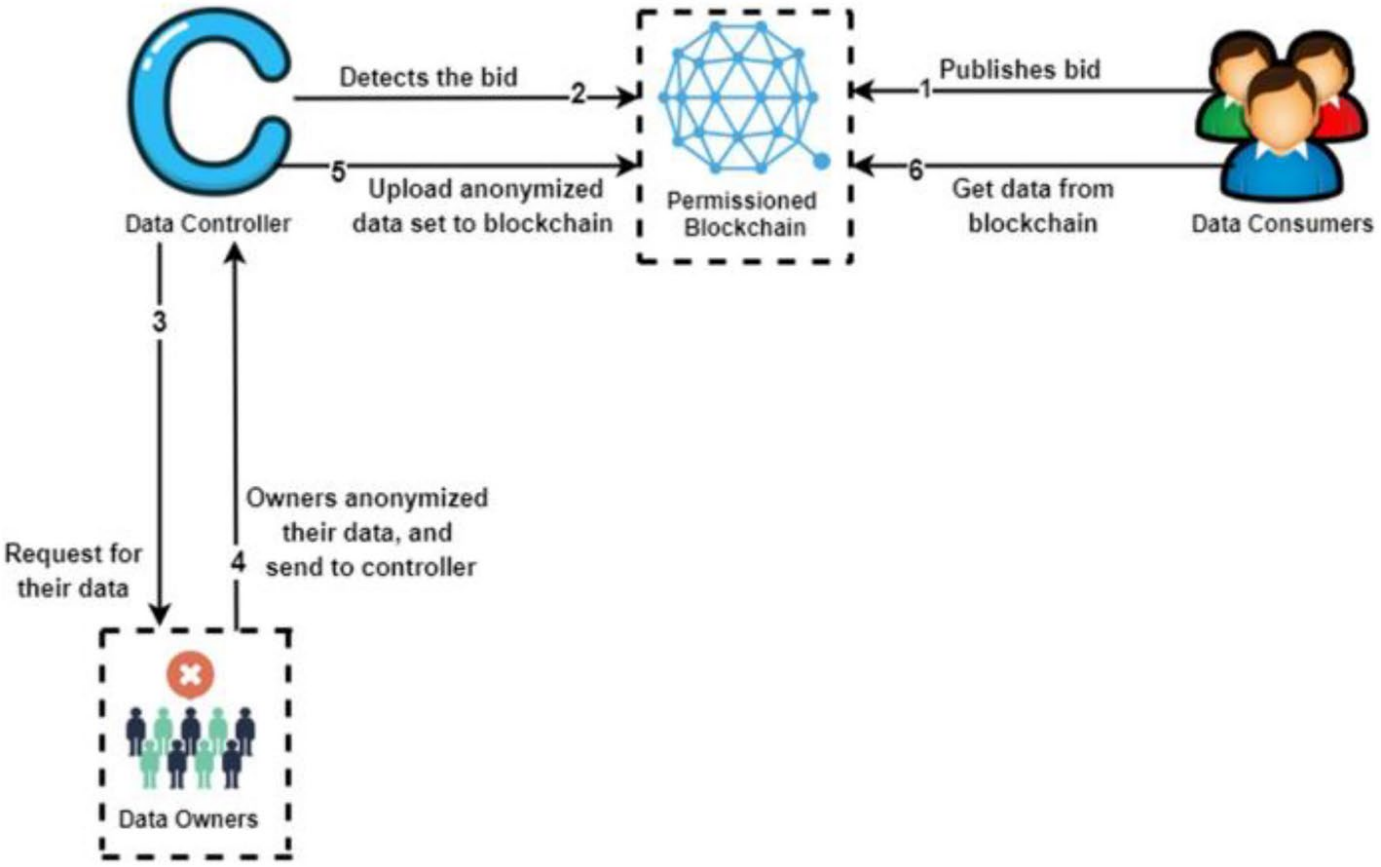
ShareChain data flow^8^.

Figure 6. shows a simplified flow for how the data sharing between data owners (patients) and data consumers (individuals or entities who seek the patients' data set) occurs.

The framework consists of four entities, data consumers, the data controller, permissioned blockchain, and data owners. The data controller (such as hospitals) is the middle entity that interacts with data consumers and data owners. Data owners share their data sets with the controller after anonymization. Data owners, consumers and controllers must register on the permissioned blockchain network to guarantee that these are authorized entities. When a data consumer requires a certain data set with some specifications, it publishes a bid on the blockchain network with these specifications. Data controllers detect these bids and communicate with the data owners to receive an offer from them. Data controllers store the offer specifications on an off-chain IPFS. Data owners use LDP for data anonymization before transferring their data sets to the data controllers. Data consumers receive the offer specifications from the IPFS and they can accept the offer and pay to the data controllers. Both data controllers and consumers establish a secure P2P communication channel to exchange identification proofs. Then, the anonymized data set of the data owners gets shared from data controllers to data consumers. Finally, data controllers complete the payment to data owners.

This framework has been implemented and tested. According to the authors, they were able to improve the latency, throughput, privacy, and accuracy compared to the benchmark model. The aim of this framework is to provide a secure and anonymized mechanism for sharing the patients’ EHRs between hospitals and consumers. However, they did not consider a mechanism for tracking patients' medical history or the mechanism of adding new EHRs to the system. They covered the sharing procedures. Thus, this framework could be part of a bigger healthcare blockchain system that aims to solve the healthcare challenges that are mentioned in the previous section.

Table 1 shows a comparative analysis of the related work with the techniques used, purposes and limitations.

**Table 1:** A comparison among related work.

Ref#	Techniques	Purpose	Limitations
A	Clusters of private Blockchains and Cloud Storage	Remote patient monitoring	Suffers from centralization issues
B	PoA Permissioned Blockchain and Smart Contracts for access control	Platform for patients and medical service providers for data sharing	Puts control in the patient’s hand so that he can add EHRs himself and still in its early stages
C	PoI Permissioned Blockchain with Merkle Tree-based for data validation and Cloud Storage	Mobile platform for sharing EHRs with healthcare providers	Puts control in the patient’s hand so that he can add EHRs himself and did not discuss the synchronization step with the cloud storage
D	Clusters of private Blockchains connected to a consortium Blockchain	Dealing with IoT devices for collecting EHRs for certain patient	Suffers from centralization issues and still a novel model
E	Permissioned Blockchain with LDP and FL along with an IPFS	Sharing patients’ data sets in a secure and anonymous way	Did not describe how the EHRs will be collected from patients to the system

## Proposed federated blockchain system (FBS)

This section aims to overcome these healthcare EHRs challenges by designing and implementing a federated blockchain system that can serve the healthcare requirements and reduce their issues.

To build a healthcare blockchain system, a decision must be made to answer the following questions:

- What is the suitable blockchain type that fulfills the needs of the healthcare industry such as a high transaction rate with high security?^18^.
- What is the appropriate consensus mechanism that serves the system requirements and reduces the scalability issues?^19^.
- What is the appropriate blockchain interoperability mechanism that enables healthcare organizations to share patients’ records among each other?^20^.

Our system design relies on the comparative study that has been conducted in Ref.^21^ to answer these questions.

The proposed FBS aims to make a global wide blockchain healthcare system that provides a unique identity for each patient across the world. Also, the system is seeking to provide interoperability not just between local healthcare providers, but also between global healthcare organizations by sharing and communicating patient’s data between hospitals. The rules that regulate data communication are governed by the protocols between each country. Finally, the system tries to link the medical Internet of Things (IoT) devices that are used to collect medical records either inside hospitals or at home using our proposed blockchain networks. Since healthcare records are critical and should not be available publicly to everyone, the system will use permissioned blockchains that are being federated by authority healthcare organizations across the globe.

**Figure 7:**
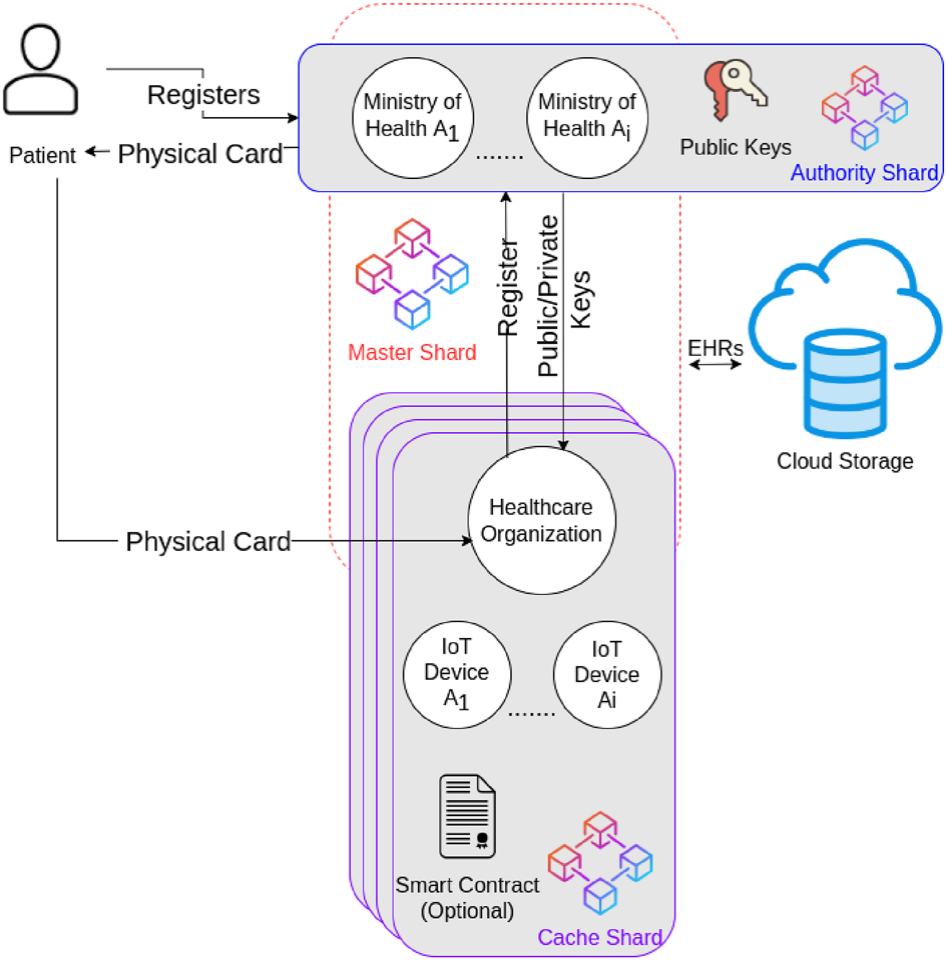
The architecture of the FBS.

The proposed FBS divides the blockchain system into modules. Each module is called a shard. The shard is basically a blockchain network that contains a subset of the data blocks of the major blockchain^22^. The FBS is constructed by combining multiple shards together. It consists of three shards; Authority, Cache and Master shards as shown in Fig. 7. Each shard contains a set of entities as participants in the network. Every entity has specific roles to perform.

In the following part, the description of each entity and their predefined roles in the network is provided.

1. Patients: They are the main actors in the system because EHRs are collected from them. They register on the system and get approval from the high level healthcare organizations to join the network. Once a patient is approved, he receives a physical card that represents his unique patient ID and contains his private key as well. Any EHR which belongs to the patient will get recorded and signed using the private key which is contained inside a physical card. Using this unique patient ID, the healthcare organizations will be able to track the medical history of any patient inside the system.
2. Patient physical ID card: After the patient registers on the system and receives approval from the authority organizations, a public–private key pair is generated for that patient. The private key is provided to the patient in the form of a physical card. Not only the physical card represents the private key, but also represents the patient's unique ID in the system that will be used to sign his EHRs.

The physical card is similar to the Automatic Teller Machine card (ATM Card) which is used to access the user's bank accounts and allow him to see transaction history or deposit an amount to his account^23^. The patient's physical card performs similarly in our system. For example, through the physical card, hospitals will be able to access the patient's medical history and will be able to append new EHRs to his medical records. The appended EHRs will be signed using the private key that exists inside the physical card before being appended to the blockchain to guarantee data authenticity and integrity.

3. High level healthcare organizations (authority organizations): They are a preselected set of healthcare organizations that will be responsible for managing the system. These organizations will be decided by each country. For example, the Ministry of Health of Egypt, U.S. Department of Health and Human Services and World Health organization…etc. These organizations will be the backbone nodes of the blockchain network and the network must launch at least using a subset of them.

They are responsible for approving patients' registrations on the system and arranging the delivery of physical cards for the patients that acts as their private key that will be used later to sign their EHRs before adding them to the blockchain.

The authority healthcare organizations are also responsible for approving the registrations of mid-level healthcare organizations such as hospitals and insurance organizations.

The public keys for both patients and the mid-level healthcare organizations are maintained by the authority organizations to be able to verify the authenticity of the collected EHRs.

4. Mid-level healthcare organizations: They represent medical service providers who interact directly with the patients to provide medical services or treatment such as hospitals, healthcare institutes, insurance companies…etc. These organizations need to register on the system and go into a preliminary approval process to join the blockchain network. The authority organizations are responsible for approving the registrations of these organizations. Upon approval, every mid-level healthcare organization will receive a public–private key pair which they will use to sign the data records.

The mid-level healthcare organizations are the entry point of EHRs into the system. Every separate mid-level healthcare organization will create a small blockchain network called “Cache shard” that will contain its medical IoT devices. These IoT devices will collect the EHRs from patients and store it inside the cache shard to be available for real-time processing. Later, the mid-level healthcare organization will transfer the collected EHRs to the Master Shard to be shared with other healthcare organizations.

For example, a hospital will register on the system. Once it is approved, it will receive its public–private key pair and establish its own cache shard. The patient will provide his physical card to this hospital to receive his medical treatment. The hospital will attach medical IoT devices to the patient to collect some measures such as heart rate or blood sugar level. These collected records will be signed by the private key that exists on the patient’s physical card and recorded inside the cache shard to be processed by doctors or other IoT devices to provide the necessary treatment for the patient. Later, when the patient completes his treatment, the hospital will sign the recorded EHRs using its private key and transfer it to the master shard to be shared with other hospitals.

5. Medical IoT Devices: They are responsible for sensing and collecting medical records from the patients. They are part of the cache shard and belong to a specific mid-level healthcare organization. These IoT devices are usually attached to the patients as wearable devices either in hospitals or at home. For example, a medical device that monitors blood sugar level or heart rate and sends the collected records to the cache shard to be processed.
6. Smart contracts: They are basically self-executing contracts that trigger an automatic action when specific conditions are met inside the blockchain network. In our proposed FBS, the mid-level healthcare organization can extend the cache shard functionality by including extra smart contracts. For example, a hospital may require adding a smart contract to the cache shard to automatically trigger an alert to the insulin pump if the blood sugar level goes above the normal level.
7. Cloud storage: It is used to store the actual EHRs. This is because the number of healthcare records is huge. Also, the size of the records could be large. Storing EHRs records directly on the blockchain is not practical because the data will be replicated to every participant node inside the network which will require each node to have huge storage to handle all the collected data inside the network. Also, storing EHRs directly on the blockchain could lead to scalability issues. Thus, cloud storage is necessary to store the EHRs while the hash of the EHRs will be stored on the blockchain to be able to verify and authenticate the data of the cloud storage. Any change to the EHRs on the cloud storage will be detectable because the hash of the altered data will not match the stored hash on the blockchain.

The next part provides detailed information about the different shards that exist in the proposed FBS.

### Authority shard

The authority shard is the top level blockchain that contains the set of high level healthcare organizations which are known as “Authority Organizations” such as the ministry of health of each country. It is the main authorization level of the system.

**Figure 8:**
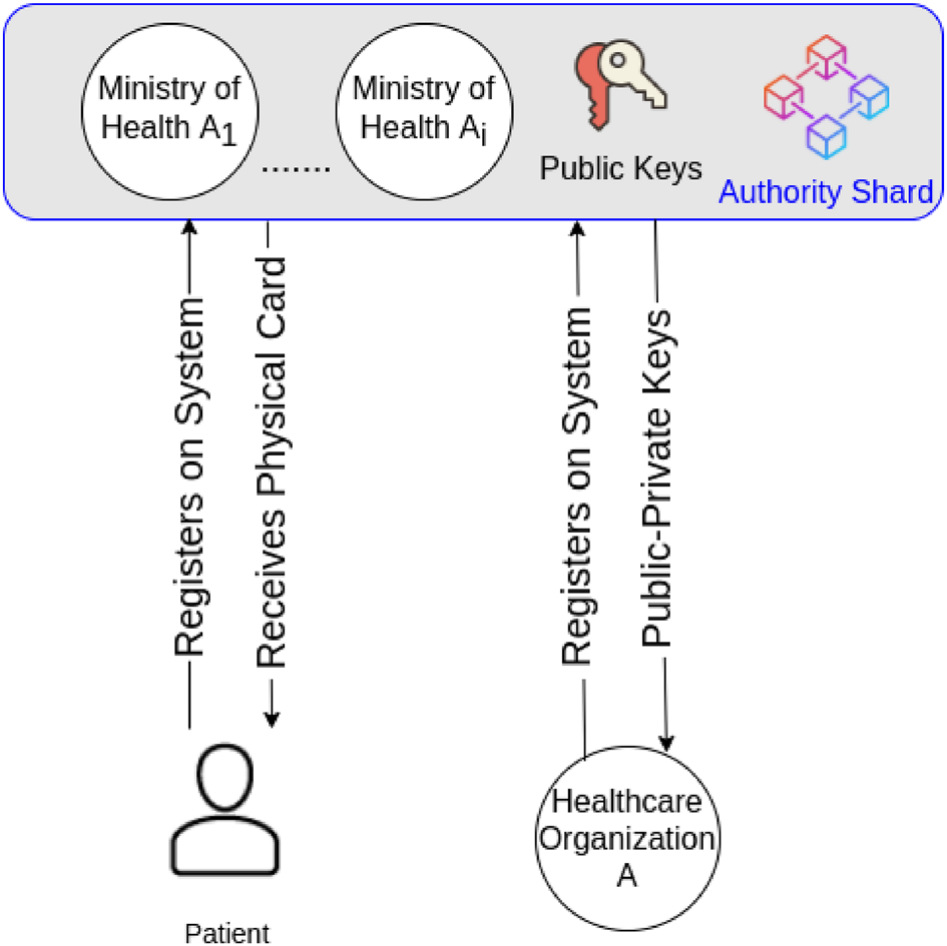
Authority shard.

When a patient registers on the system, he receives approval from the high level healthcare organizations to join the blockchain network. Once the patient is approved, a public–private key pair is generated for that patient (see Fig. 8). The public key is saved into the authority shard and the patient receives a physical identity card that acts as his private key which is used to sign his medical records before being added to the blockchain. The public key is used to verify the authenticity of the data for that patient.

The same scenario is done with the mid-level healthcare organizations. They register on the system and get approval from the high level healthcare organizations. They receive a public–private key pair and the public key is saved in the authority shard.

The mid-level organizations are usually granted permissions based on their role in the system. For example, hospitals may receive read and write permissions, while insurance companies may receive read permissions only.

The authority shard is a delay tolerant network since patients who register on the network must wait until they receive the physical identity card. Then, it will use Proof of Stake (PoS) as a consensus mechanism in which the coins will be distributed over the high level healthcare organizations. The nodes will use their stake to mine the incoming transactions. PoS consensus has proven results to improve the scalability of the blockchain network and it does not consume energy as discussed in Ref.^24^.

### Cache shard

The cache shard terminology is inspired from the Central Processing Unit (CPU) cache memory in which the data is stored temporarily for processing, and then stored in the main memory. The cache shard acts as a temporary storage that carries the EHRs collected from a patient during receiving a specific service or treatment to be available for real-time processing. This is necessary to eliminate any emergency case that could endanger the patient’s life.

**Figure 9:**
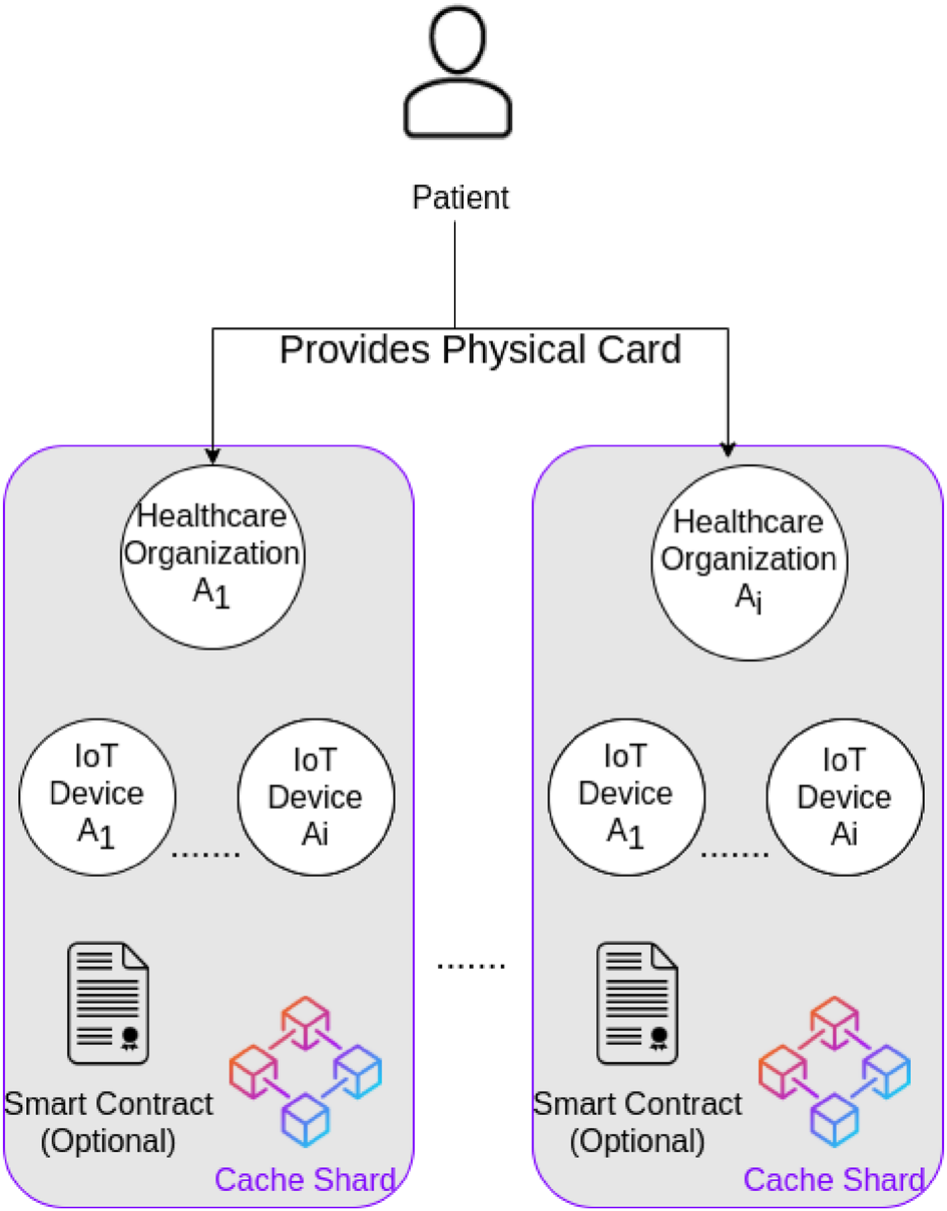
Cache shard structure.

The cache shard is a separate blockchain network. It is usually a blockchain network within a specific healthcare organization. It is a low level private or consortium blockchain network that contains a mid-level healthcare organization as a validator. Additionally, this blockchain may contain IoT devices that collect EHRs from the patients. The patient provides his physical identity card to the mid-level healthcare organizations where his collected EHRs get signed using his private key and signed once again using the organization's private key before being added to the blockchain. Figure 9 clarifies the cache shard architecture.

The most important aspect of this shard is that it accelerates the speed of transaction approval. This is why it is using private or consortium blockchain type to benefit from the high transaction speed of these types of networks.

Additionally, this shard may include smart contracts as an optional feature to perform some automated actions in the network if specific conditions are met. These smart contracts can be used in conjunction with IoT devices to enable communication between them or to automatically perform an emergency action that could save the patient’s life based on the reading information coming from his EHRs. The cache shard structure is flexible because it will be used within a specific healthcare organization such as a hospital. The type of the cache shard is restricted to private or consortium blockchain to increase the security of this network and also because the EHRs are not public data that should not be accessed by anyone.

The choice of the consensus mechanism in this layer is open to the healthcare organization. For example, if a hospital requires real-time data processing, then it will need a very efficient consensus mechanism with a high transaction rate.

Since the cache shard is a permissioned blockchain, which means that any node will go into a preliminary process before joining the blockchain network, we recommend Proof of Authority (PoA) as a consensus mechanism for this level of blockchain. PoA is best suited for permissioned blockchains networks and it has a high transaction rate^25^. Additionally, it overcomes the scalability issues. Thus, healthcare organizations can benefit from these advantages inside their cache shard.

### Master shard

**Figure 10:**
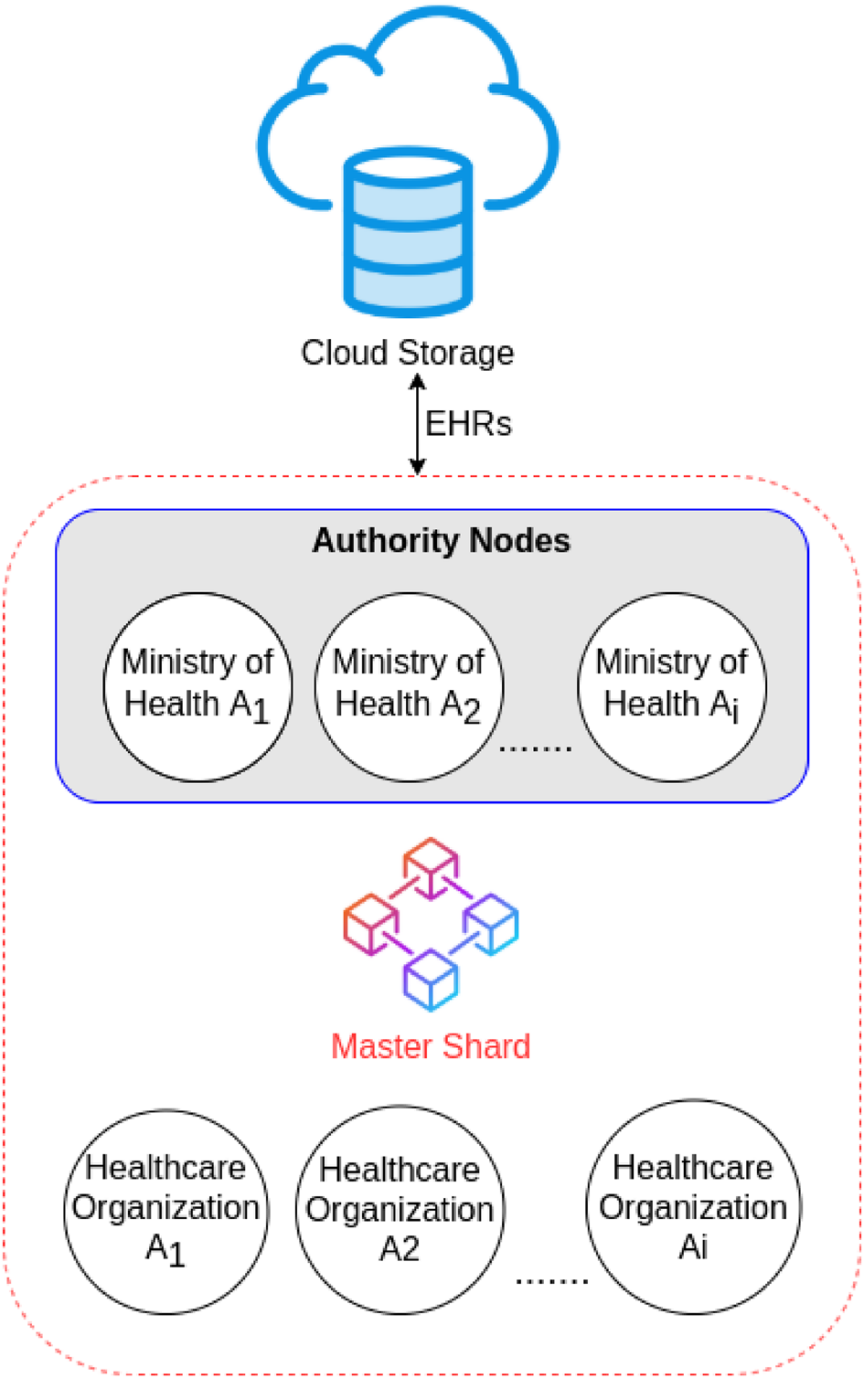
Master shard.

The master shard is a consortium blockchain that contains the mid-level healthcare organizations and is being federated by the high level healthcare organizations (see Fig. 10).

All of these organizations have gone through a verification process to be verified entities inside the network. Additionally, the number of nodes inside this shard is expected to be huge which will make this blockchain prone to scalability issues and transaction latency. Thus, PoA will be used as a consensus mechanism to reduce the expected scalability problems and improve the transaction rate in the master shard.

The cache shards are used to collect and process the EHRs in real-time to overcome the emergency case. Later, the mid-level healthcare organizations transfer these collected EHRs to the master shard such that they can be shared and accessed by other mid-level healthcare organizations as other hospitals and healthcare institutes if the patient decides to receive the medical care there.

One of the main benefits of the master shard is to allow interoperability between healthcare organizations and solve the data blocking issue in which hospitals refuse to share the patient’s data.

*What if a hospital does not transfer the patients’ data to the master shard?* In order to overcome this issue, a reputation rank will be attached to every mid-level healthcare organization. Once a mid-level organization joins the network, it receives a predefined reputation rank which identifies the organization as a new node. This reputation rank increases as a reward every time a mid-level healthcare organization transfers the patient’s data to the master shard. Mid-level healthcare organizations with low reputation rank can be considered by the authority organizations and could be removed from the network as a consequence for not transferring the medical records to the master shard. This mechanism will encourage all mid-level healthcare organizations to transfer the patients’ data to the master shard to gain high reputation and stay as a part of the network to benefit from accessing the patients’ medical history (based on his request) and improve its medical care service.

Since the number of nodes in the master shard will be large, this network may suffer from some transaction latency. Latency will be acceptable for this blockchain because it does not involve any emergency handling.

The EHRs may contain large data. Thus, this blockchain will save the EHRs in secure cloud storage and only the hash of this data will be stored in the master shard. The hash is used to verify that the data stored in the cloud storage is not tampered or changed in any way.

To improve the security of this shard and to provide a mechanism to detect a malicious node, a reputation rank will be attached to every node. The reputation rank increases as the node produces valid blocks that are being verified by the other nodes inside the network. When a node gets attacked and starts producing false data, it will be detected by the other nodes and that node will lose its reputation and protective procedures can be done to remove that node from the network until it recovers from the attack. After that, the node can re-join the network again and start building its reputation once again.

Since the proposed FBS consists of multiple shards, these shards must be able to communicate with each other to transfer the EHRs from one shard to another based on the modular structure that has been shown in Fig. 7. The interoperability between blockchains has always been a fatal problem. Numerous researches have been conducted in this area as discussed in Refs.^7,26,27^. The most popular interoperability platforms are Cosmos and Polkadot. Both of them came to production recently^28^.

In the proposed FBS, we used Cosmos platform to build our system shards and to benefit from its interoperability mechanism. A detailed discussion of how Cosmos platform enables blockchains interoperability can be found in Ref.^21^.

**Figure 11:**
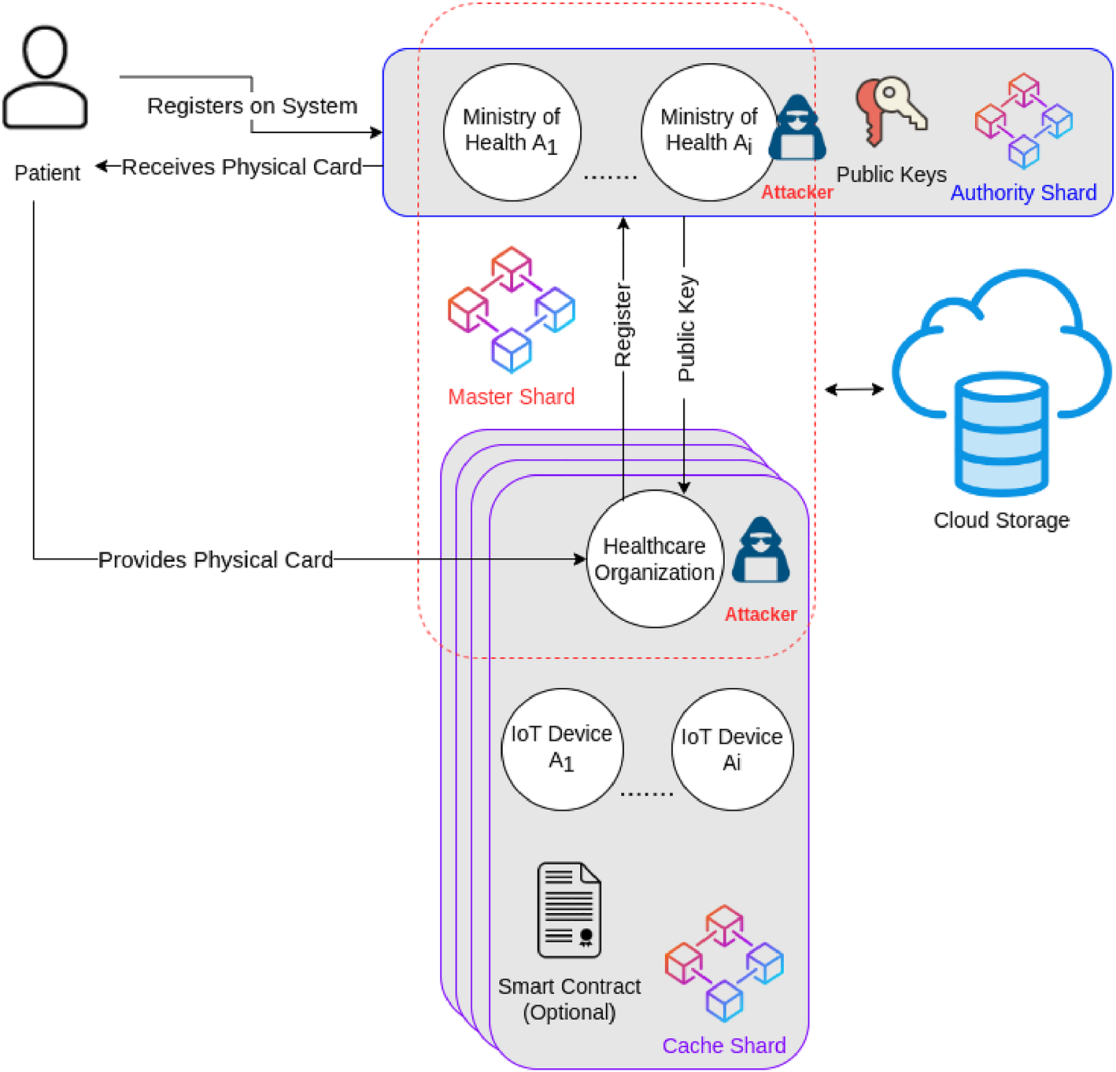
Attacker model of the FBS.

We have examined the points of attack in the FBS. Figure 11 shows the attacker model of our proposed system.

Both patients and healthcare organizations register on the system by sending their data to the authority nodes. One or more of the authority nodes could be prone to attacks. However, to overcome this issue we used a consortium blockchain for the authority shard. Thus, the decision of completing the registration of patients and healthcare organizations does not depend only on a single authority organization. Another threat is that the healthcare organization node which the patient is given his trust to deal with his data could be prone to attack. Our model suggests using consortium blockchain for the cache shard as well to reduce the effects of such a threat.

## System validation

Since healthcare medical records are sensitive data, they are not available publicly. Thus, to validate the federated system we used random data sets as follows:

- Random patients’ information
- Random healthcare organizations
- Random data hashes for the EHRs using Secure Hash Algorithm 256 (SHA256).

Unfortunately, we have no available distributed systems cluster. Therefore, the tests are done on a local machine with the following specifications:

CPU: Intel Core I7 4th Generation 2.4 GHz 8 Cores.

- RAM: 8 GB DDR3
- Graphics: Nvidia GeForce GTX 950 M
- Hard Drive: 1 TB Samsung Evo 860 SSD
- Operating System: Ubuntu 20.04.3 LTS 64-bit.

Our performance analysis is concerned about two measures; average speed of different transactions and how scalability affects the transaction speed.

To execute the queries and messages during our tests, we have developed a shell script for each type of the validations. This script repeatedly performs a certain query or message multiple times to measure the average time of executing that action and by taking into account the increase of the records in the system.

### Validation of adding patients to the authority shard

We measured the speed of adding 10, 100, 1,000, 5,000, 10,000, and 50,000 patients to the federated blockchain system on multiple stages. In each stage, we increased the number of patients to see how the system performs.

**Figure 12:**
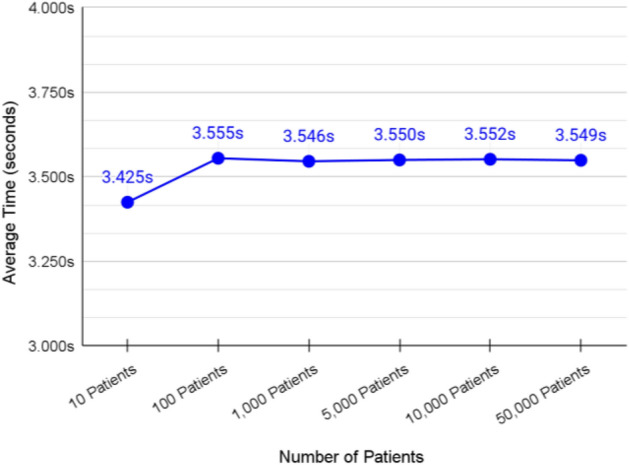
Average transaction speed of adding patients to authority shard.

The average transaction speed of adding patients to authority shard was around 3.530 s as shown in Fig. 12.

The transaction speed was not affected much when the number of patients increased in the system. This is due to the powerful mechanism that Cosmos uses for handling storage as nested key-value stores which improves the performance even when the system scales.

### Validation of querying a specific patient from the authority shard

**Figure 13:**
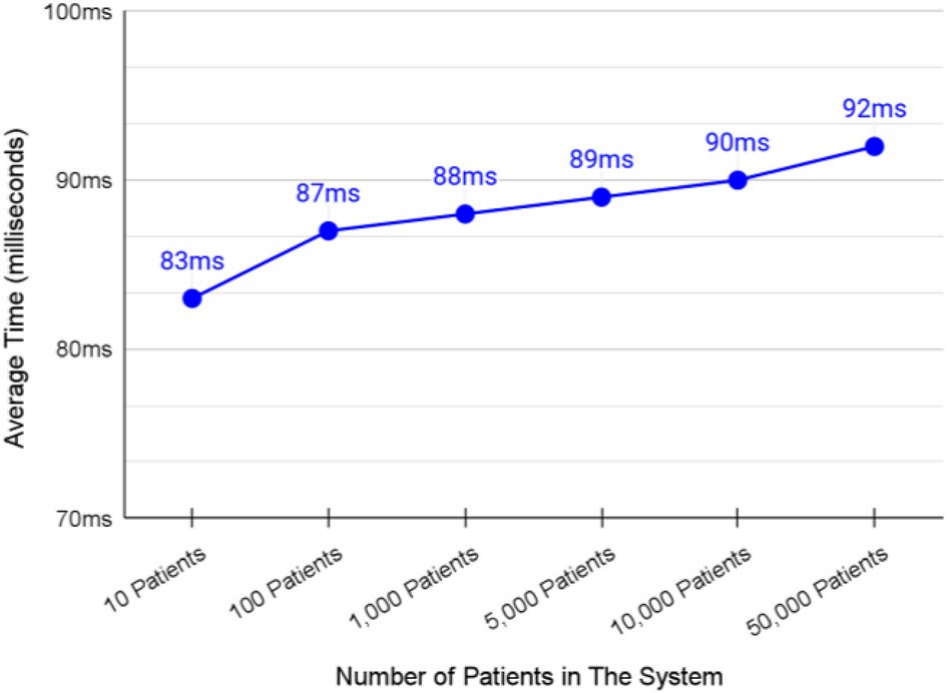
Average transaction speed of querying a single patient from authority shard.

The average transaction speed of querying a single page of patients from the federated blockchain chain was about 88 ms as shown in Fig. 13. The speed of querying patients was not affected much by the increasing number of blocks in the system. These results are suitable for the proposed system.

### Validation of adding mid-level healthcare organizations to the system

**Figure 14:**
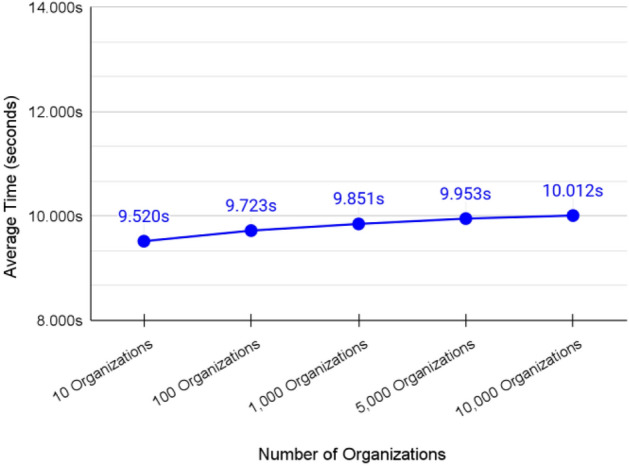
Average transaction speed of adding organizations.

The average transaction speed of adding a new single healthcare organization to the system was about 9.8118 s as shown in Fig. 14.

The transaction speed was not affected much by the increasing number of organizations. However, we were able to reduce the average time to 4.231 s by adding a continuous sequence of organizations. Cosmos IBC optimized that sequence by grouping packets together which reduced the handshaking steps of the IBC protocol. For example, adding 10 organizations in a separate way will require 10 packets which will perform the handshaking protocol 10 times. On the other hand, when adding 10 organizations as a sequence, Cosmos was able to group them into 3 packets which in turn reduced the handshaking protocol to 3 times. Therefore, the overall time required to process the transactions is reduced. The grouping mechanism depends on the state of the network and the Cosmos relayer.

Definitely, extra overhead is expected when switching to the production environment due to the networking part of sending and receiving packets between the authority and master shard. However, this part of the system tolerates delay as it does not involve any emergency actions that could endanger the patient’s life.

### Validation of adding EHRs to cache shard

**Figure 15:**
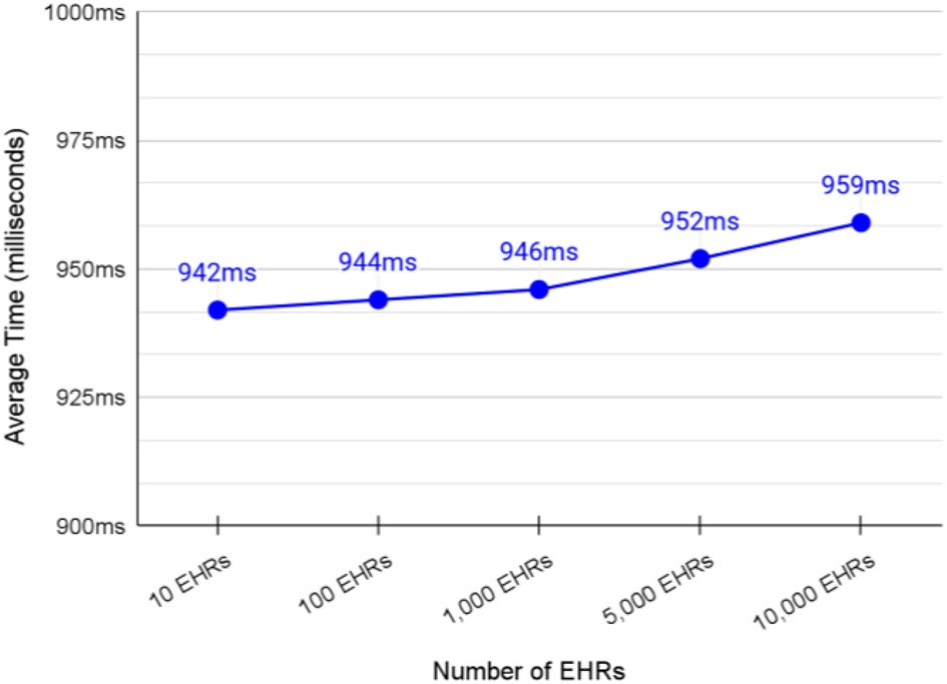
Average transaction speed of adding a specific patient’s EHR to the cache shard.

The average transaction speed of adding a new EHR to the cache shard was about 947 ms (see Fig. 15). This rate must be kept as low as possible because any delay at this shard may endanger the patient’s life. Most of the cache shards can run locally to guarantee low rate.

### Validation of querying EHRs of a specific patient from the cache shard

We measured the speed of querying EHRs from the cache shard by querying EHRs pages. A page of EHRs contains up to 10 EHRs depending on the offset at which the page starts and the available number of EHRs after that offset. In our testing, we always query the most recently added EHRs. Thus, we target the last EHRs page.

**Figure 16:**
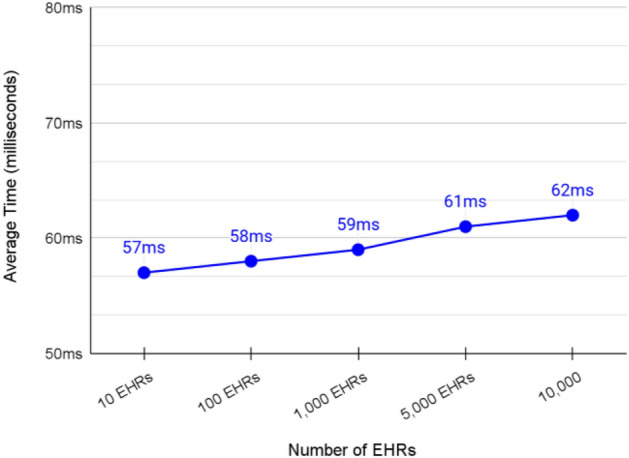
Average transaction speed of querying the latest EHRs page from the cache shard.

The average transaction speed of querying a single page of EHRs from the cache shard was 59 ms as presented in Fig. 16. This rate is acceptable for the purpose of this shard. In addition, the average transaction speed was not affected by increasing the number of EHRs in the cache shard. According to these results, we believe that the cache shard can be scalable in production environments.

### Validation of transferring EHRs from the cache shard to the master shard for a specific patient

**Figure 17:**
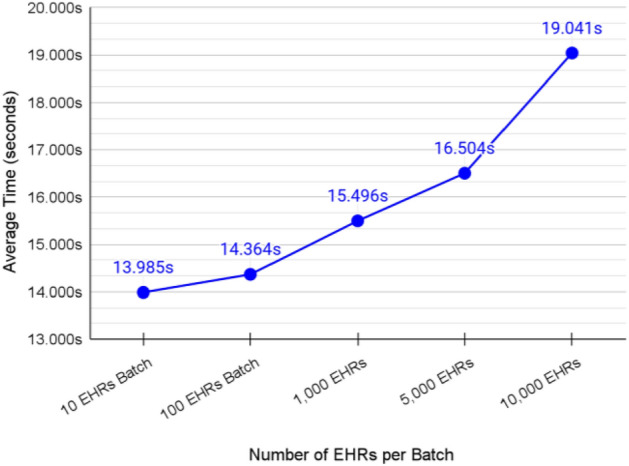
Average transaction speed of transferring a batch of EHRs.

The average transaction speed of transferring a single EHRs batch from the cache shard to the master shard was about 15.878 ms as shown in Fig. 17. The higher the number of EHRs per batch, the lower the transaction rate per EHR. Simply, this happens as larger batches require less number of packets to be transferred because the EHRs are grouped together which minimizes the overhead of Cosmos IBC handshaking protocol between shards as discussed previously. However, sending over 10,000 EHRs per batch may fail. The reason for that is that Cosmos assigns a gas fee per transaction. This gas fee can be decided during configuring the relayer. Once the gas runs out, the transaction is rejected. The gas fee protects the networking from receiving large transactions that could freeze the chain.

### Validation of querying a specific patient EHRs from the master shard

**Figure 18:**
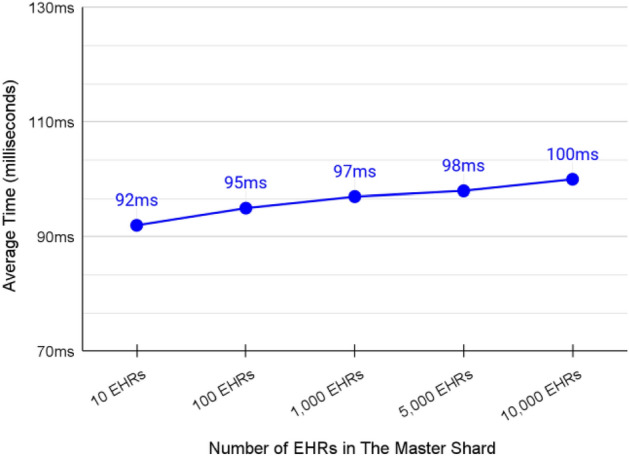
Average transaction speed of querying the latest EHRs page from the master shard.

The average transaction speed of querying a single page of patients from the FBS was about 96 ms as shown in Fig. 18. The speed of querying EHRs was slightly affected by increasing the number of blocks in the system. However, it is still suitable for satisfying the purpose of the master shard.

### System validation summary

**Figure 19:**
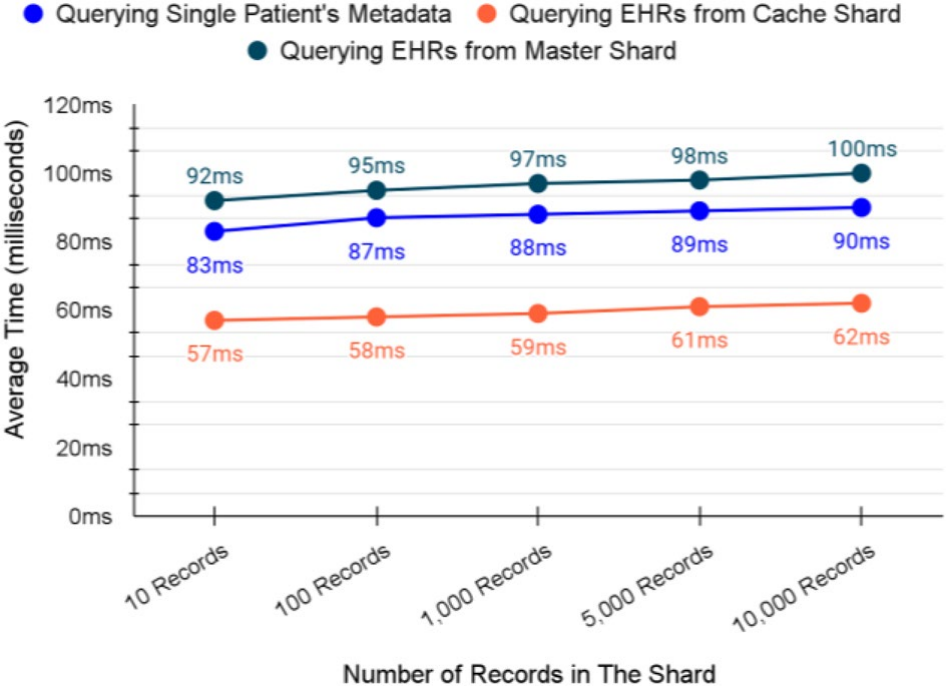
Queries validation in milliseconds.

The analysis divided the transactions into two types; Queries and Messages. Queries are responsible for retrieving data, while Messages are responsible for adding or transferring data in one of the shards of the proposed FBS. Figure 19 validation the performance of the queries.

**Figure 20:**
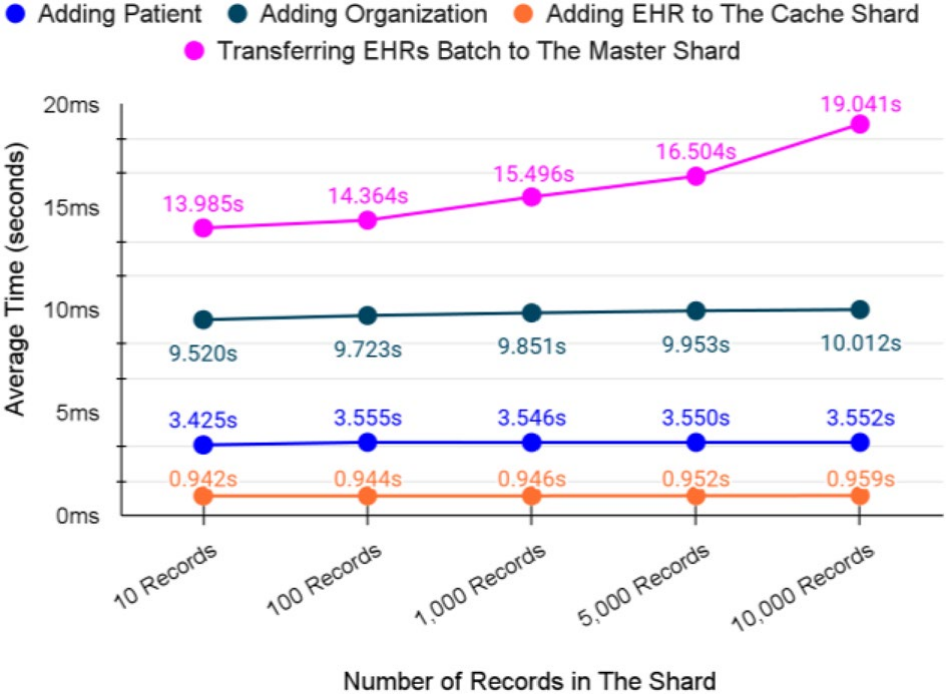
Messages validation in seconds.

Figure 20 summarizes the validation of the messages.

The validation of both adding and querying from the cache shard consumes less time compared to the other operations of the same type. That is one of the main requirements of the proposed FBS as the cache shard serves emergency cases. Also, other adding and querying operations provide promising results as they almost remain stable even when the number of records in the system is increased. Thus, we believe that our proposed FBS can perform similarly in the production environment and can provide the same scalability.

## Conclusion and future work

Healthcare is a critical industry that is suffering from security, interoperability and EHRs management issues which can endanger patients’ lives or cause death. The work in this paper discussed these issues in detail and proposed a blockchain system that is being federated by authority healthcare organizations. The system aims to provide a mechanism to store the medical history of patients while giving them the ability to share it with medical service providers during receiving medical treatment. The FBS is divided into three shards; Authority Shard, Master Shard and Cache Shard. The system uses Cosmos platform to enable interoperability among these shards. Each shard has certain roles and contains a subset of the information on the system. Also, the effectiveness of the system has been validated which showed an average of 68–100 ms for performing query operations and average of 0.944–19.041 s for performing writing operations. Finally, a discussion of the system limitation has been provided.

For future work, many different adaptations, tests, and experiments have been left for the future using real data on a distributed system cluster. Also, a complete web application as an interface for registration of both patients and organizations will be built using our federated blockchain.

We are very curious about enhancing the cache shard architecture as it is the front door that deals directly with patients’ treatment including emergency cases. In this paper, we provided a very basic implementation for the cache shard to be able to collect EHRs for patients. We are interested in improving the cache shard by including smart contracts and testing its performance.

Lastly, we are going to investigate the integration between our proposed federated blockchain system and an InterPlanetary File System (IPFS) or a cloud middleware which manages a cloud storage that will hold the actual data to guarantee data integrity.

## Data Availability

The FBS source code is available from the corresponding author on reasonable request at the following links https://github.com/ashraf-mohey/authority, https://github.com/ashraf-mohey/master and https://github.com/ashraf-mohey/cache. For the datasets, we generated random records and used them as input to the system for testing purposes. Thus, we did not use any real patients’ data. We confirm that all experimental protocols were approved by the Faculty of Computers and Artification Intelligence—Cairo University. We confirm that all methods were carried out in accordance with relevant guidelines and regulations.
